# Power Factor Compensation Using Teaching Learning Based Optimization and Monitoring System by Cloud Data Logger

**DOI:** 10.3390/s19092172

**Published:** 2019-05-10

**Authors:** Antonio Cano Ortega, Francisco Jose Sánchez Sutil, Jesús De la Casa Hernández

**Affiliations:** Department of Electrical Engineering, University of Jaen, 23071 EPS Jaen, Spain; fssutil@ujaen.es (F.J.S.S.); jcasa@ujaen.es (J.D.l.C.H.)

**Keywords:** Power factor compensation monitoring system PFCMS, power factor PF, Cloud data logger CDL, Teaching learning based optimization TLBO, capacitor bank optimization CBO

## Abstract

The main objective of this paper is to compensate power factor using teaching learning based optimization (TLBO), determine the capacitor bank optimization (CBO) algorithm, and monitor a system in real-time using cloud data logging (CDL). Implemented Power Factor Compensation and Monitoring System (PFCMS) calculates the optimal capacitor combination to improve power factor of the installation by measure of voltage, current, and active power. CBO algorithm determines the best solution of capacitor values to install, by applying TLBO in different phases of the algorithm. Electrical variables acquired by the sensors and the variables calculated are stored in CDL using Google Sheets (GS) to monitor and analyse the installation by means of a TLBO algorithm implemented in PFCMS, that optimizes the compensation power factor of installation and determining which capacitors are connected in real time. Moreover, the optimization of the power factor in facilities means economic and energy savings, as well as the improvement of the quality of the operation of the installation.

## 1. Introduction

Differently from residential loads, most commercial and industrial premises have a high uptake of inductive loads such as electric motors, inductive/resistor load, sodium vapour, and metal halide lighting, etc. These installations and operation of these devices distort power supply and reduce power factor. Normally, a lower power factor for facilities causes a huge amount of losses and may lead to a thermal problem in switchgear. Fortunately, power factor is controllable with properly designed power factor improvement methods.

At present, society is involved in a major digital transformation due to major advances in information and communication technologies. This digital transformation extends to the technological sector, offering very interesting development possibilities. New technologies developed include the Internet of things, big data, cloud computing, industry 4.0, and intelligent networks.

The application of new technologies to industry 4.0 allows monitoring the operating of the system and controlling the system with computers and mobile devices remotely. In this sense, the measurement and monitoring of electrical variables is interesting for studying the energy behaviour of installations. Excessive reactive energy consumption implies an increase in the electricity bill, so it must be reduced to achieve significant energy and economic savings.

To achieve energy and economic savings by correcting the power factor of the installation, it is necessary to solve an optimization problem. There are many optimization algorithms applied to the resolution of engineering problems, such as the Genetic Algorithm (GA), Particle Swarm Optimization (PSO), Artificial Bee Colony (ABC), etc. The most commonly used evolutionary optimization technique is the genetic algorithm (GA). However, GA provides a near optimal solution for a complex problem having a large number of variables and constraints. Parameters such as population size, crossover ratio, mutation ratio are difficult to determine, this being the main difficulty in applying this algorithm, which influences the effectiveness of the algorithm. Similarly, PSO and ABC require others configuration parameters.

The main reason to use an optimization algorithm is its capacity to solve different optimization problems effectively and efficiently. In this paper, TLBO is proposed to obtain global solutions for continuous non-linear functions with less computational effort and high consistency. The TLBO method is based on the effect of the influence of a teacher on the output of learners in a class.

Rao et al. [1] compared different optimization methods, which shows the advantage of using the TLBO algorithm over other optimization algorithms (GA, PSO, ABC, etc.); different experiments have been carried out to test the effectiveness of the TLBO against other optimization techniques, with different objective functions.

The TLBO algorithm applies in [2], developing a new advanced TLBO algorithm to process parameter optimization in selected modern machining. In electrical systems, TLBO is used to solve different problems, such as obtain the optimal power flow (OPF) by means of a non-domination based sorting multiobjective presented in [3]. In [4,5,6], the TLBO approach is used to minimize power loss and energy cost by optimal placement of capacitors in radial distribution systems.

Moreover, there are different technologies for power factor correction (PFC), an overview of the state of the art in reactive power compensation technologies, the principles of operation, design characteristics of reactive power compensators implemented with thyristors, and self-commutated converters are presented in [7]. Another paper [8], realizes an analysis and compensator design framework for power-factor compensation based on cyclodissipativity. The challenges and power quality issues faced in the micro grid are proposed in [9] by a review of compensation methods that achieve an improved power factor and real power balance from the load point of view. The technical and economic value of power factor improvement, verified through an analysis of a real-world electrical system and loads, is implemented in [10].

Numerous investigations have been done on PFC in electrical installations. In this sense, in [11] a new digital PFC control strategy overcomes the problem of limited switching frequency due to a limited digital signal processor speed. In [12] a Fuzzy logic controlled synchronous motor is used for reactive power compensation. A fuzzy logic controlled synchronous motor can give a very fast response to the reactive power required by the load. Another approach to compensate for reactive energy is studied in [13], that uses a Programmable Logic Controller (PLC) based PFC method for a 3-phase Induction Motor (IM) through switching of shunt capacitors. In this context [14], the correction method use an intelligent PFC based on PLC as a control system especially for correction of the industrial power factor.

Other PFC methods develop different compensation systems, for example in [15] a static var compensator prototype was used to test and validate a variety of control strategies. An automatic PFC unit was developed in [16], which is able to monitor the energy consumption of a system and automatically improve its power factor, the device calculates the reactive power consumed by a system’s inductive load and compensates the lagging power factor using capacitance from a capacitor bank (CB).

In AC/DC conversion systems it is necessary to compensate the power factor. The paper [17] analyses a control algorithm of a three-phase three-level PFC rectifier, using a mathematical analysis of two-level space vector modulation. In [18], a system for reactive power compensation for battery/photovoltaic hybrid power source of hybrid electric vehicle in real time is presented. In this context, a predictive algorithm for AC–DC three-phase converters with active PFC is presented in [19].

The normative section includes [20,21,22], the IEEE Standards 18-2012, C37.26-2014, and C37.99-2012, in which are developed the IEEE standards for shunt power capacitors, the protection of shunt CB´s, and the methods of power-factor measurement for low-voltage inductive test circuits.

A multitude of papers have emerged utilizing smart meter data, as a new smart voltage and current monitoring system technique proposed in [23,24], as well as a smart home electric energy saving system implemented by combining smart meter, smart plug, smart mobile devices, and database server. The meters measure consumption on a very fine scale, usually on a 15 min basis, and the data giving unprecedented granularity of consumption patterns at household level is analysed in [25].

This paper presents the development and implementation of PFCMS, using the open source Arduino platform where the TLBO algorithm is implemented to operate in real time. Therefore, the interest of the proposal is based on the advantage of the Arduino platform with a highly efficient algorithm such as TLBO, in order to reduce the calculation and operation times of PFCMS.

In the first phase, the design of a CB involves determining the number and capacity of the capacitors. For this, there should be a set of measurements of the electrical variables of the installation and a comprehensive analysis of the installed loads. In this research, the algorithm CBO developed calculates the optimal capacity of all capacitors to install. The number of capacitors influences the complexity of the problem decisively, in this sense to evaluate the size of the capacitors, TLBO algorithm reduces simulation times. CBO algorithm uses TLBO as the basis for optimization. The CBO algorithm has to be programmed in a high-level language, such as C, Python, MatLab, etc. This research uses MatLab as a programming environment.

The second phase, starting from the results of the optimal capacity to be installed, the compensation of the power factor is done using TLBO in real time. As proof, there was analysis with PFCMS: a series of tests were performed with different types of loads, such as lamps, motors and resistive-inductive loads, where the electrical variables of the installation are measured and monitored, using the sensors installed in PFCMS. Once the electrical variables were obtained, the TLBO algorithm was applied that determined the combination of capacitors at each moment in order to obtain the power factor nearest to the desired value. The experimental results demonstrate the feasibility of the proposal.

The measurement time can be adjusted from 0.2 to the desired time. Moreover, reduced measurement time, together with the speed of resolution of TLBO, allows using PFCMS in real time. Therefore, TLBO complies with the definition of the problem to be solved in this investigation.

The design of a CB involves determining the number and capacity of the capacitors. For this, there should be a set of measurements of the electrical variables of the installation and a comprehensive analysis of the installed loads. In this research the algorithm CBO developed calculates the optimal capacity of all capacitors to install. The number of capacitors influences the complexity of the problem decisively, in this sense to evaluate the size of the capacitors, TLBO algorithm reduces simulation times. CBO algorithm uses TLBO as the basis for optimization. 

It is possible to integrate the PFCMS with Android applications to be used with mobile devices under this operating system. It is specially designed to work with an app developed for this type of device. The apps for Android devices have been developed with the free programming environment of Google MITAI [26].

All software development has been done with free programming environments, so that the system can be reproduced by any researcher without the need to acquire commercial software licenses.

To make the measurements of the electrical variables, the electric power meter PZEM-004t (PZEM) [27] (Ningbo Peacefair Electronic) was used. The measured variables are voltage, current, active power, and energy, from which reactive, apparent power and PF are derived, for system monitoring.

The aim of this work is to design and implement a power factor compensation system using a TLBO algorithm in real time, additionally developing an algorithm to obtain the optimal capacity of the CB, monitor the system in GS, and a smartphone application to monitor the installation in real time. PFCMS has been designed to correct power factor in different electric systems. The paper is organized as follows: Section 2 presents the relevant related theory power factor, algorithm TLBO, and optimal capacity algorithm, Section 3 deals with the hardware and software development of PFCMS. Section 4 defines the case studies and results. Finally Section 5 exposes the conclusion of the present investigation.

## 2. Theoretical Background

### 2.1. Introduction to Compensation Power Factor

Power factor is the ratio between the active power *P* and the apparent power *S* in an electrical load. It is simply a measure of how efficiently the load current is being converted into useful work output. The lower the power factor of a system, the less economically it operates. A low power factor can be the result of a significant phase difference between voltage and current at load terminals.

Generally, it is the use of inductive loads such as IM, power transformers, induction furnaces, and so on that causes a current to lag behind voltage. A poor power factor resulting from inductive loads can be improved by power factor correction method. Since power factor in inductive loads is generally lower, they have to be supplied with reactive power in order to reduce increased power consumption of the facilities.

All inductive loads require *P* to perform the actual work, and reactive power *Q* to maintain the magnetic field. This *Q* is necessary for the equipment to operate, but imposes an undesirable weight on the supply, causing the current to be out of phase with the voltage (current lags the voltage). Low power factor can also result when inactive motors operate at less than full load, etc.

PFC is applied to neutralize as much of the magnetizing current as possible and to reduce losses in the distribution systems [28,29,30]. A new approach for real time voltage control of distribution networks that has improvements over the conventional voltage control models is [31]. It offers many benefits to the commercial electrical consumer, including reduced utility bills by eliminating charges on reactive power, reduced losses making extra *S* available from the existing supply. Thus, it improves energy efficiency.

### 2.2. Compensation Power Factor Theory

The design of the device for compensating the adverse effects of inductive *Q* is a crucial task in designing any PFC systems. The capacitive current supplied by the capacitors is directly connected across the industrial load terminals or electrical installation. The consumer advises to improve the power factor beyond the value of magnetizing kVAr rating of the load [14].

Lower power factors can dramatically increase the required current being consumed by an appliance to work correctly. The following equation calculates the amount of reactive power that is wanted to be produced by the bank of capacitors connected in parallel to the load:

(1) $$Q_{C}=P\times [\tan(\varphi_{V}-\varphi_{I})-\tan{\left(\varphi_{V}-\varphi_{I}\right)}^{\prime }]$$

where *φ_V_* is the angle of the voltage signal and *φ_I_* is the angle of the current signal, and *Q_C_* is the reactive power the capacitor must provide; and (*φ_V_* − *φ_I_*)′ is the angle we want for the power factor once it is corrected.

This involves a detailed survey of loads connected to the system and performing measurements of the electrical variables of the facilities during that period that allows us to determine the optimum capacity of the CB. The *Q_C_* requirement value changes with the load variation, hence the algorithm control developed such that the reactive power requirement near the load terminals is maintained at a constant by varying the capacitive reactive power and the power factor is maintained nearer to unity.

Knowing that the resistance of a capacitance is dependable on the frequency, it is known that the capacitance needed to correct a certain amount of reactive power in single phase is

(2) $$C=\frac{Q_{C}}{2\times \pi \times f\times V^{2}}$$

where *C* is the capacitance in Farads, *f* is the frequency, and *V* is the voltage.

In the three-phase systems, it can be connected to CB in wye (W) and delta (D). In the case of the wye connection, the voltage phase to phase *V_PP_* is the voltage per phase by the square root of 3, and the equation will be

(3) $$C_{\lambda }=\frac{Q_{C}}{2\times \pi \times f\times V_{PP}^{2}}$$

In the three-phase system connected in delta, the voltage phase to phase is equal to the voltage per phase, the equation will be

(4) $$C_{\Delta }=\frac{Q_{C}}{3\times 2\times \pi \times f\times V_{PP}^{2}}$$

### 2.3. TLBO Algorithm

TLBO is a global stochastic optimization algorithm, based on populations and oriented to large-scale problems. It is based on modeling the behavior of a class of students formed by a set of known candidate solutions [32].

These solutions are progressively improved by simulating both the teaching process of a teacher and the interaction between students. The main characteristic of TLBO is its performance and its lack of specific search parameters, since it is only necessary to specify the population size and the number of cycles as required.

To achieve this, TLBO relies on two fundamental steps per cycle, the phase of the teacher (Teacher Phase (TP)) and that of the students (Learners Phase (LP)).

The TP difference between the respective mean result of each subject and the corresponding result of the teacher for each subject is

(5) $$X_{new}=X_{i}+r\times \left(X_{teacher}-T_{F}\times X_{mean}\right)$$

where, *X_teacher_* is the best individual, *X_i_* is other individual, *X_mean_* is the current mean of the individuals, *T_F_* is the teaching factor which decides the value of mean, and *X_new_* is the influence for student *X_i_* by the difference between the teacher´s knowledge and the qualities of all students.

In the LP, two possible solutions are selected, each final individual *i* from the previous stage is paired with another, different from himself, *j*. Next, we try to move the individual *i* from his current position in a direction that depends on its relative value with respect to *j*, finally, the individual *i* will only be updated if his value is improved after the change.

(6) $$X_{new}=X_{i}+r\times \left(X_{i}-X_{j}\right)$$

(7) $$X_{new}=X_{i}+r\times \left(X_{j}-X_{i}\right)$$

#### Problem Formulation

The objective of optimal PFC problem of electrical installations is to optimize the objective function, defined as the difference between power factor desired (PF_DES_) and power factor obtained (PF_OBT_) while satisfying all operational constraints.

The optimization function used to obtain the compensation of the power factor in real time is given by

(8) $$F=\left|\cos\left(\varphi_{des}\right)-\cos\left[\tan^{-1}\left(\frac{q\left(t\right)-\left(\sum_{j}^{n}\sum_{i=0}^{1}C_{j}*i\right){\times 2\times \pi \times f\times v(t)}^{2}}{p(t)}\right)\right]\right|$$

where cos(*φ_des_*) is the PF_DES_, *q*(*t*) is the reactive power, *p*(*t*) is the active power, and *v*(*t*) is the voltage of the installation for a time *t*, *C_j_* are the capacities of the capacitors and *f* is the frequency of the installation.

The constraint used in the formulation of the problem for the developed TLBO algorithm has been taken:

(9) 0.95≤cosφ≤1

PFC depend on the current legislation in each country. Each country has intervals defined between 0.9 and 0.95 as a minimum value to apply surcharge for excessive reactive energy consumption, in Spain it is 0.95, this value is set for the constraint. The maximum constraint value is set to 1, because if the limit is exceeded the power factor changes from inductive to capacitive.

The limit values of the constraints can be adjusted to the legislation in each country, or to the needs of the installation where the PFCMS is implanted.

To define the population (*pop*), 2^*n*^ possible states are considered, where *n* is the number of capacitors used. Each state shows the combination of turning on or off the n capacitors.

(10) $$pop=\left[\begin{array}{ccc} X_{1,1} & \cdots & X_{1,n}\\ \vdots & \ddots & \vdots \\ X_{2n,1} & \cdots & X_{2n,n} \end{array}\right]$$

**Figure 1:**
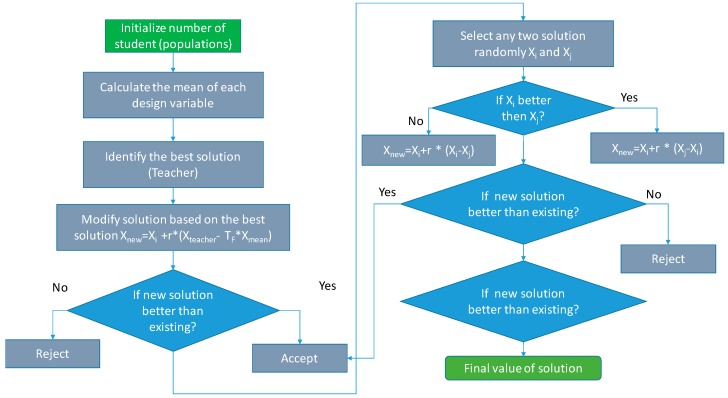
Flow chart of the teaching learning based optimization (TLBO) Algorithm.

Figure 1 shows TLBO flow chart.

This algorithm will be implemented in the PFCMS in real time. In addition, it will be used within the optimization algorithm of point 2.4.

### 2.4. CBO Algorithm

The power factor compensation study of electrical installations is necessary to perform a set of measurements of the electrical variables involved. Once the measurements have been done, the capacities of the different capacitors that formed the CB can be determined.

In this research a CBO algorithm is proposed to obtain the optimal capacity of each of the capacitors. This algorithm allows calculating a capacitors bank composed of 2 … *n* units, achieving an optimal compensation. If the number of capacitors increases, a better PF compensation is obtained, but it implies higher installation costs and a more complex control.

**Figure 2:**
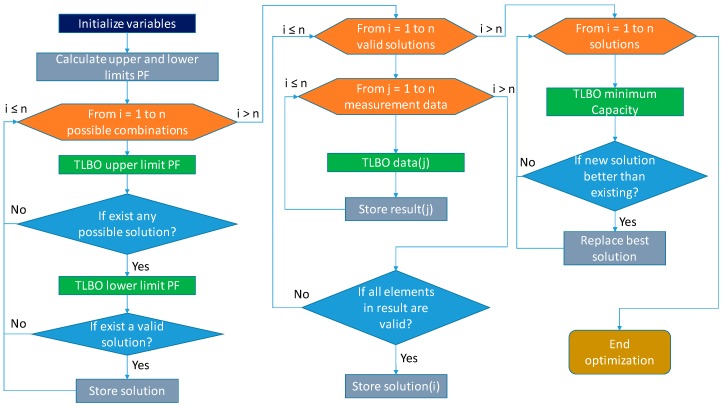
Flow chart of the capacitor bank optimization (CBO) Algorithm.

The implementation of the CBO algorithm takes place in three phases: (i) obtain the possible solutions to cover the maximum and minimum power factor, (ii) with the set of the possible solutions, choose those that make it possible to compensate the entire acquired field of measurements, (iii) with the chosen solutions, select the one with the lowest capacity. Figure 2 shows CBO flow chart.

## 3. PFCMS Design

**Figure 3:**
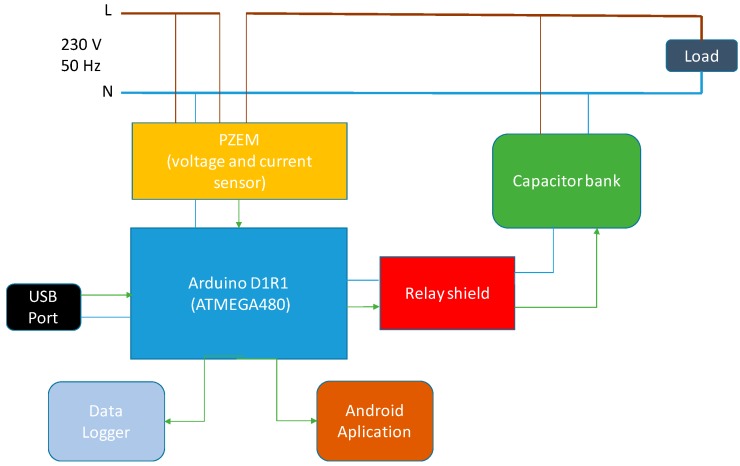
Power Factor Compensation and Monitoring System (PFCMS) scheme.

The proposed system takes 230 V 50 Hz mains supply as a power source. The sampled voltage signals and current signals receive of power grid and process through the voltage sensor and the current sensor circuit and introduce in the microcontroller, that performs power factor calculations using TLBO and switches capacitors of CB. Moreover, results are stored and monitored in CDL using GS, and can also be displayed on Android application. Figure 3 shows the functional block diagram of the complete project. A smart sensor network that allows inspecting an electrical installation in a non-intrusive way is presented in [33], developing an open-architecture smart sensor network, based on FPGA technology, which is able to continuously monitor PQ in industrial facilities, public buildings, and residential homes.

### 3.1. Hardware

#### 3.1.1. Microcontroller

A microcontroller is a small computer on a single integrated circuit containing a processor core, memory, and programmable input/output peripherals [34]. An AD1R1 microcontroller (based on ESP-8266EX) is used in this research, which has lots of libraries developed and available online for free. Table 1 shows the main characteristics of the microcontroller.

Mains voltage and current, real power, of the network is received through the developed program from PZEM. The reactive power and PF are calculated by the microcontroller. Moreover, the design of switching strategy of CBs is explained in the control algorithm, in point 2.3.

Board AD1R1 allows access to Wi-Fi networks, and therefore uploading information to the Internet, in our case to GS with times less than 1 s. It makes it ideal for the dual function of a monitoring system and uploading information to the cloud.

#### 3.1.2. Electric Power Meter

The PZEM model of the Ningbo Peacefair Electronic has been chosen as electrical meter, whose characteristics are in Table 2. This equipment can measure voltage, current, active power, and active energy. To measure the current, use a non-invasive toroidal transformer through which the phase cable passes into it.

Inside, the PZEM module has two opto-couplers (PC817A) has a connection with pins 1 and 2 of the PZEM through a voltage divider with two resistors (R1 and R2) of 1 kΩ in series (extracting only 2.5 Vdc in this point). With the insertion of a resistor (R3) of 1 kΩ in parallel with the resistance R1, a new voltage divider is obtained at that point.

### 3.2. Software

Two programs are developed: (i) AD1R1, which performs the functions of reading electrical variables from PZEM, the optimization calculations and sends the data to GS; (ii) Android application, which monitors the data stored in GS, making it possible to display industry data in real time at any location using Smartphone or Tablet.

The measurements made with PZEM can be adjusted to different measurement times depending on the needs of the electrical installation, this time is independent of the Internet upload time.

#### 3.2.1. Microcontroller Program

This part of the software should be responsible for several tasks: (i) the program must start the Wi-Fi system, the electric power meter PZEM, and the initialization of the four outputs to the relays that will activate the contactors to operate in output mode; (ii) read the electrical variables from PZEM; (iii) perform TLBO algorithm; (iv) data is uploaded to Internet.

**Figure 4:**
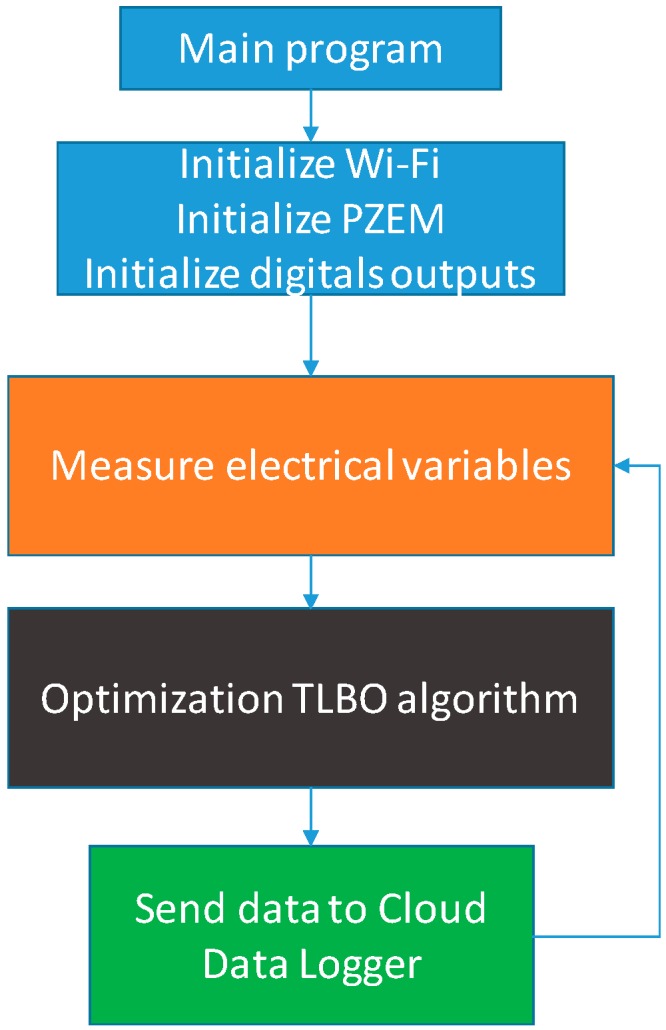
PFCSM main program.

Figure 4 shows the flow diagram corresponding to the main program.

#### 3.2.2. Android App

The program developed for Android mobile devices allows visualizing the behavior of the installation in real time.

MITAI is used as development environment. This platform is free and available from Google for the online development of apps for Android devices.

The app developed with MITAI consists of two parts: (i) the development of screens using visual components such as buttons, lists, text boxes, and others, and non-visual components such as BT, Wi-Fi, etc.; (ii) programming through blocks that allows the development of the application with all the functionalities.

**Figure 5:**
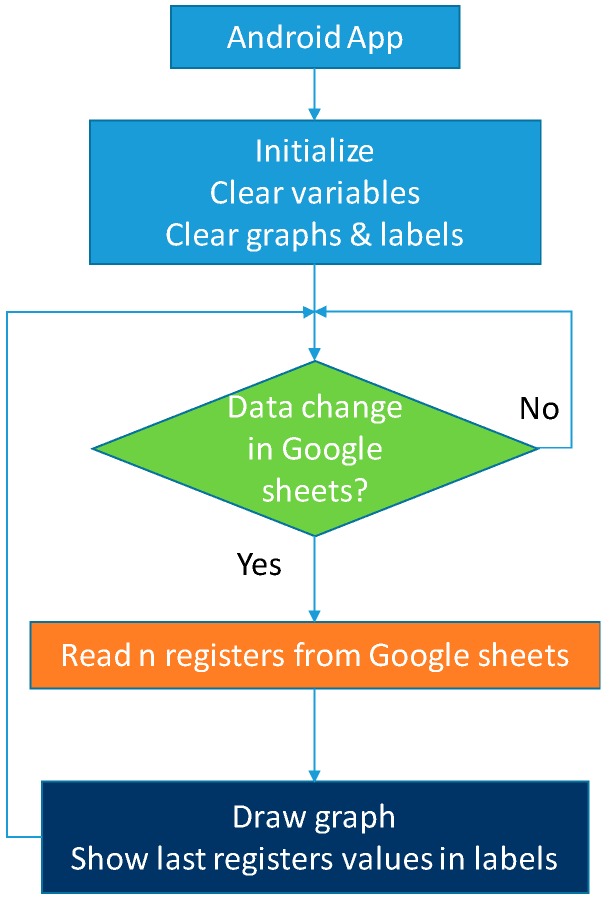
PFCMS Android app flow diagram.

Figure 5 shows the flow diagram of the application for mobile devices.

### 3.3. Prototype

PFCMS built has external connections, each one connects with the capacitor. The communication between AD1R1 and Internet integrated inside the microcontroller, which allows data to be uploaded from the board, without the need for other external components.

Moreover, equipment is powered by an external adapter that provides the necessary voltage to AD1R1. There are other multiple ways of feeding the equipment, which can be implemented without any difficulty in other similar projects.

It is possible to work with contactors whose coil has a maximum current of 10 A, which is the one supported by the relays. In case of use contactors with coils whose current is greater than 10 A, it is easily realizable only by changing the relays to the next step of 30 A, which are easily found in the market.

**Figure 6:**
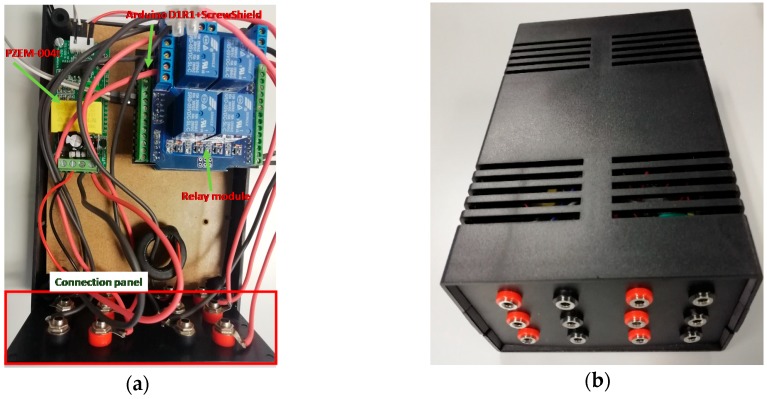
PFCMS: (**a**) Internal connections; (**b**) Final prototype.

PFCMS built, the connection of the components, the control panel and the equipment mounted in the container box is shown in Figure 6.

Programmer includes PF_DES_ and capacitors values into the microprocessor. Depending on the difference between the PF_DES_ and power factor installation (PF_INST_), the TLBO algorithm adjusts the switching schemes of capacitors banks.

Optimal solutions and measured electrical variables are stored in GS with the fixed measurement interval. The spreadsheet represents the graphs of voltage, current, power factor, and active, reactive, and apparent powers over time.

## 4. Results and Discussion

A CB of four units is used to do the tests, divided into two phases: (i) obtain the optimal capacities for the four capacitors in each case studied, (ii) with the optimal capabilities perform the tests in real time using PFCSM.

### 4.1. Test Equipment

#### 4.1.1. Test Machine

The case studies have been done in the electrical machinery laboratory of the Electrical Engineering Department of the University of Jaen. In this laboratory, there are several trainers of electrical machines, in the power range of 0.3 kW and electric machine of 1.5 kW.

This trainer has a machine that can simulate the behavior as resistant torque, speed control, and different applications such as cranes, water pumps, etc. This machine has a control panel to allow all these functions. In this case, only the resistive torque mode has been used, to obtain the different PF for each motor curves and be able to compare results.

The CB has some capacity positions and dissipate a power of up to 825 VAr.

#### 4.1.2. Used Machines

For the studies, three types of motors have been used, two squirrel cage rotors (3-phase) of 1.5 and 0.37 kW, and a squirrel cage rotor (1-phase) with star capacitor. Table 3 illustrates the characteristics of the machines used.

#### 4.1.3. Load Lamps (Electric Ballast)

For the studies, two types of lamps have been used, the sodium vapour and metal halide. Table 4 illustrate the characteristics of the lighting used.

In the [35] presents a comparative study of the operational characteristics of the HPS lamp when fed by conventional ballast versus electronic ballast.

Metal halide and sodium vapour lamps have ballast with a highly non-linear load which changes during the warm-up period.

#### 4.1.4. Resistance-Inductance Load

The loads connected to the electrical system are mostly resistive-inductive. Moreover, resistors and inductances have seven positions and a power of up to 1200 W and 900 VAr respectively. Table 5 shows the characteristics of these resistances and inductances.

### 4.2. Capacitor Bank Optimization

The measurements carried out in the different equipment studied allow obtaining the optimum capacity installed in the CB. The algorithm developed in 2.4 applies to the different loads to obtain the optimum in each case.

This algorithm is developed in MatLab, since the measurements made are very extensive, and need a longer calculation time.

The best solutions reported by this algorithm are in Table 6. As this algorithm requires a fine tuning of controlling parameters to get the optimum results, hence the results obtained by this algorithm may not be the global optimum solutions. However, to find out the optimum values of the common controlling parameters such as population size and the number of iterations, the algorithm is executed a number of times with different population sizes and number of iterations. At this point, it is important to clarify that in the TLBO algorithm, TP updates the solution as well as in the LP.

Table 6 shows the range of capacities, the possible solutions, the optimal solutions, and the best solution for the time interval analysed, in each of the tests. Each test has {[max(C_i_)–min(C_i_)]/step}^n^ maximum solutions.

With the optimal solution obtained for each case of Table 6, algorithm 2.3 applies to verify that the solution is correct in the range of measurements made.

In addition, Table 7 provides the statistical results of objective function obtained by different loads, in which all the performance indices of TLBO are shown. Here, the initiation sets are included in Table 6. Table 7 shows the average, standard deviation, maximum and minimum difference between PF_OBT_ and PF_DES_, of the best solution obtained for the measurements made in each case.

### 4.3. Compensation Power Factor Test in Real Time

The energy supply companies increase the electricity bill through a surcharge for excessive consumption of reactive energy. The usual values are surcharge below 0.95 and without surcharge between 0.95 and 1. PF_DES_ to perform the tests is 0.95.

Different loads used are motors, lamps (ballasts), and resistive and inductive loads. This equipment performs the measurements of the PFC tests and analyses the data obtained in real time.

PFCMS has implemented the algorithm described in point 2.3 to obtain the optimal combination of capacitors installed in the CB.

The optimum capacity is defined by the Equations (3)–(5), if CB is connected in single-phase, three-phase, in star or triangle, respectively. In addition, value obtained cannot be adjusted to a combination of capacitors with standard capacities existing on the market. The capacitance obtained is the capacitance selected in the CB at each moment by using TLBO.

Moreover, the graphs for each of the tests carried out show the difference between the calculated optimum capacity and the capacity obtained by TLBO in PFCMS to control the CB.

#### 4.3.1. Motors Load

Three scenarios are defined to perform this test.

1. Motor 0.37 kW (3 phase). Delta D connection.
2. Motor 0.37 kW (3 phase). Wye W connection.
3. Motor 0.37 kW (1 phase).

The capacity values used for TBLO Algorithm are 0.5-0.5-0.5-0.5 μF for the three-phase motor in D and W, obtained from CBO algorithm shown in Table 6.

The torque changes every 40 s from no load to 0.6 Nm with a step of 0.1 Nm, with the test machine. The acquisition of data has been done with PZEM configured to measure each 1 s.

**Figure 7:**
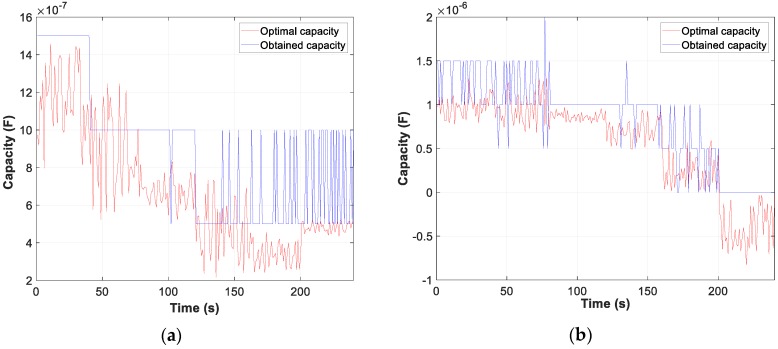
Comparison obtained vs optimal capacity: (**a**) Motor in W; (**b**) Motor in D.

**Figure 8:**
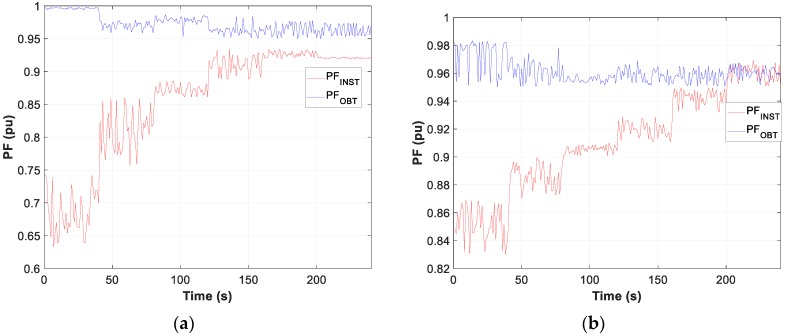
Comparison of power factor installation (PF_INST_) vs power factor obtained (PF_OBT_): (**a**) Motor in W; (**b**) Motor in D.

**Figure 9:**
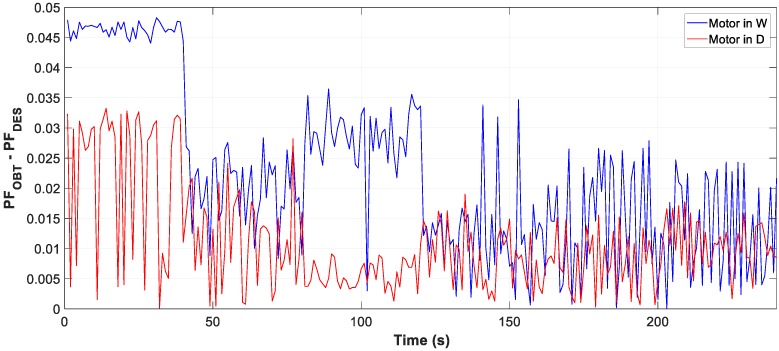
Difference between PF_OBT_ and power factor desired (PF_DES_).

Moreover, the test results of motor D and W connection, in Figure 7, Figure 8 and Figure 9, show the evolution of the obtained vs optimum capacity, PF_INST_ and PF_OBT_, and the difference between PF_OBT_ and PF_DES_, for 1 s application.

**Figure 10:**
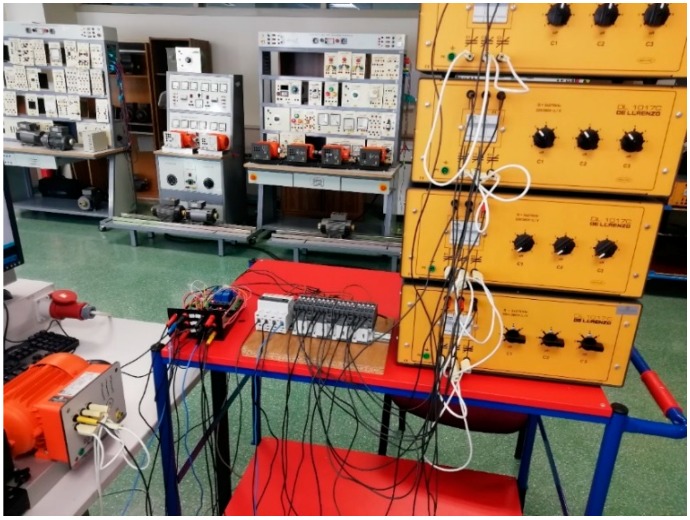
Final assembly for the study of the three phase motor load.

Figure 10 illustrates the assembly done in the laboratory.

The capacity values used for TBLO algorithm are 0.5-0.5-1-2.5 μF for the single-phase motor, obtained with CBO algorithm are in Table 6.

**Figure 11:**
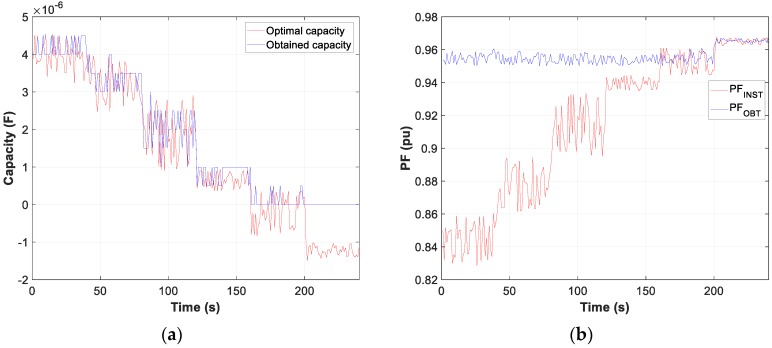
One-phase motor comparison: (**a**) Obtained vs optimal capacity; (**b**) PF_INST_ vs PF_OBT_.

**Figure 12:**
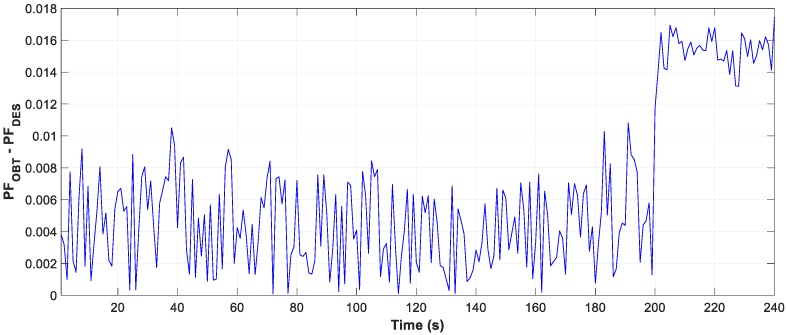
Difference between PF_OBT_ and PF_DES_ (1-phase motor).

The test results of motor one-phase, shown in Figure 11 and Figure 12, are the evolution of the obtained vs optimum capacity, the PF_INST_ and power factor obtained (PF_OBT_), and the difference between PF_OBT_ and PF_DES_, for 1 s of TLBO algorithm application. Figure 11a shows a PF_INST_ greater than 0.95, with an optimal capacity less than zero and the obtained capacity equal to zero.

The difference between PF_OBT_ and PF_DES_, for three-phase motors in D and W, is lower than 0.0332 and 0.0483 respectively, and for the single-phase motor it is lower than 0.0174. The results obtained indicate that TLBO algorithm is applicable to this type of problems.

#### 4.3.2. Lamps Load

The facilities use a variety of lamps for lighting. This test utilizes sodium vapour and metal halide lamps. In order to perform this test, it defines two scenarios.

1. Metal halide lamp.
2. Sodium vapour lamp.

The tests illustrate the process of starting the lamps up to the permanent operating mode. The starting time is approx. 2 min. Moreover, graphs show 240 s of operation, the permanent regime reach to 120 s. The capacity values used for TBLO algorithm are 5-5.5-7-8 and 5-5-7-8 μF for metal halide and sodium vapour respectively, obtained from CBO algorithm show in Table 6.

**Figure 13:**
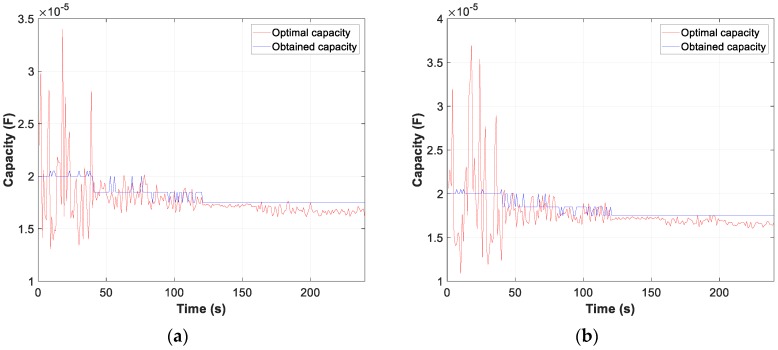
Comparison obtained vs optimal capacity: (**a**) Metal halide; (**b**) Sodium vapour.

**Figure 14:**
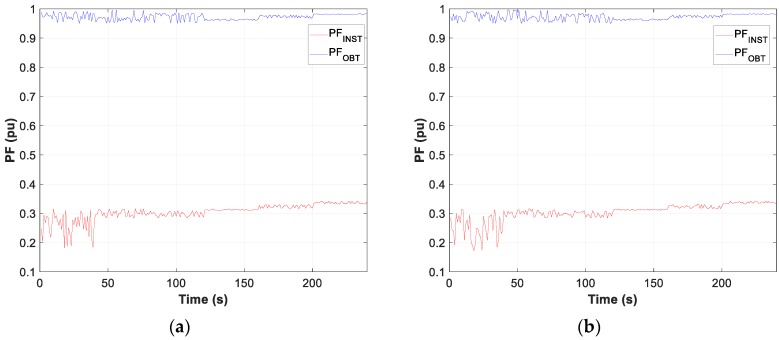
Comparison PF_INST_ vs PF_OBT_: (**a**) Metal halide; (**b**) Sodium vapour.

**Figure 15:**
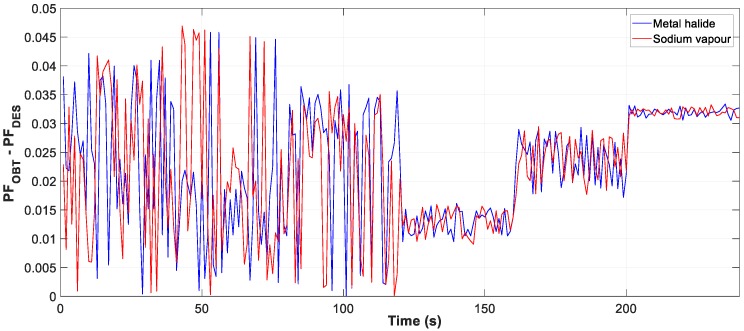
Difference between PF_OBT_ and PF_DES_ (Metal halide).

Figure 13, Figure 14 and Figure 15 show the evolution of the obtained vs optimum capacity, PF_INST_ and PF_OBT_, and the difference between PF_OBT_ and PF_DES_ for different lamps, with an application time of 1 s for TLBO algorithm.

The difference between PF_OBT_ and PF_DES_, for sodium vapour and metal halide lamps, is less than 0.0458 and 0.0469 respectively. The results obtained indicate that the TLBO algorithm is applicable to lighting installations.

**Figure 16:**
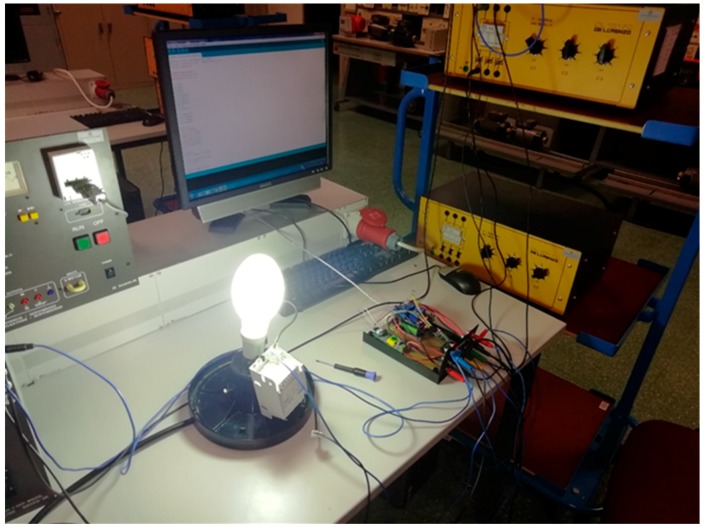
Final assembly for the study of the lamps load. Sodium vapour.

Once the assembly is done in the laboratory, it remains in the shape shown in Figure 16.

#### 4.3.3. Resistance and Inductance Load

In order to do this test, it defines two scenarios, in which the load was taken resistance fixed and the inductance change. In this case, the defined scenarios are as follows:

1. Resistance-Inductance series load
2. Resistance-Inductance parallel load

Tests show the different operating states of the loads. The reactance variation time is 40 s approximately. Moreover, capacity values used are 0.5-2-4-7.5 and 0.5-1.5-2.5-2 μF, for parallel and series respectively, obtained from the CBO algorithm show in Table 6.

**Figure 17:**
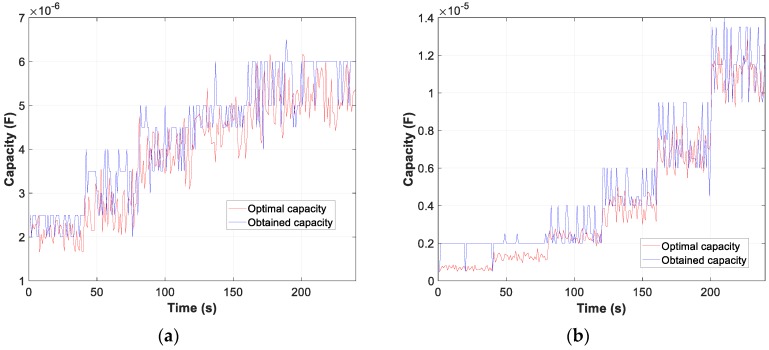
Comparison obtained vs optimal capacity: (**a**) Series connection; (**b**) Parallel connection.

**Figure 18:**
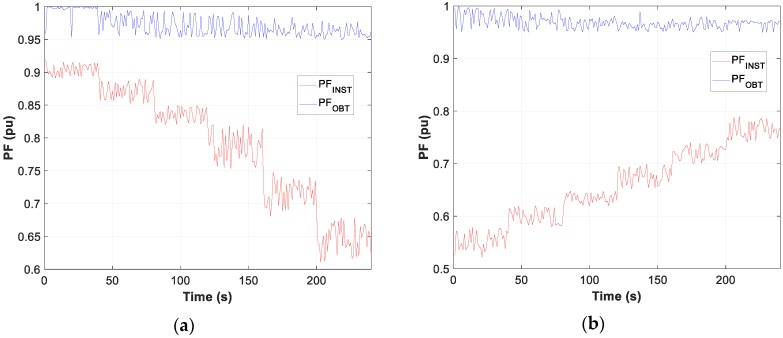
Comparison PF_INST_ vs PF_OBT_: (**a**) Series connection; (**b**) Parallel connection.

**Figure 19:**
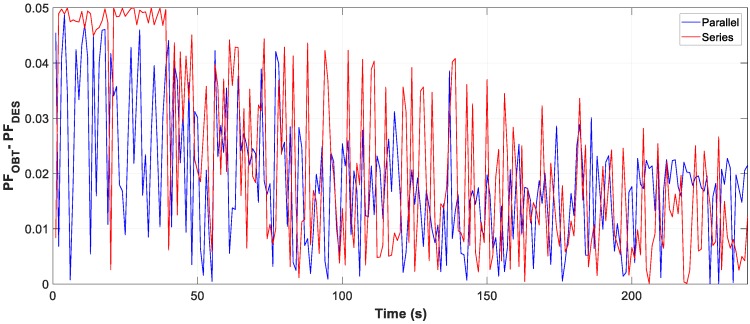
Difference between PF_OBT_ and PF_DES_.

The test results reflect in Figure 17, Figure 18 and Figure 19, the evolution of the obtained vs optimum capacity, PF_INST_ and PF_OBT_, and the difference between PF_OBT_ and PF_DES_, with an application time of 1 s for the TLBO algorithm.

The difference between PF_OBT_ and PF_DES_, for series and parallel loads, is less than 0.050. The results obtained with the TLBO algorithm indicate the correct operation for different loads.

#### 4.3.4. Daily Use of IM

Data used correspond to one day (86,400 s) of 1.5 kW motor operation. The capacity values used for TBLO algorithm are 1.5-10-9.5-6.5, obtained from the CBO algorithm show in Table 6.

**Figure 20:**
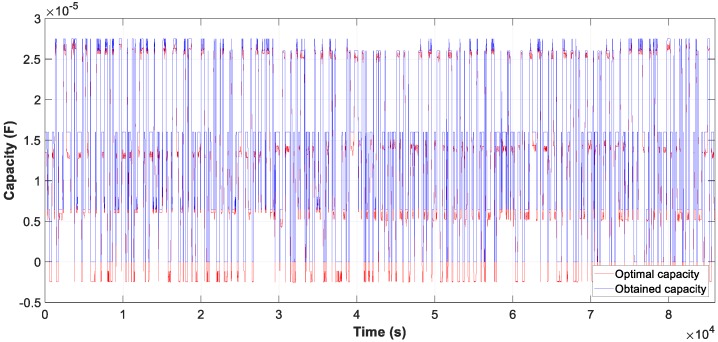
Comparison obtained vs optimal capacity of daily.

**Figure 21:**
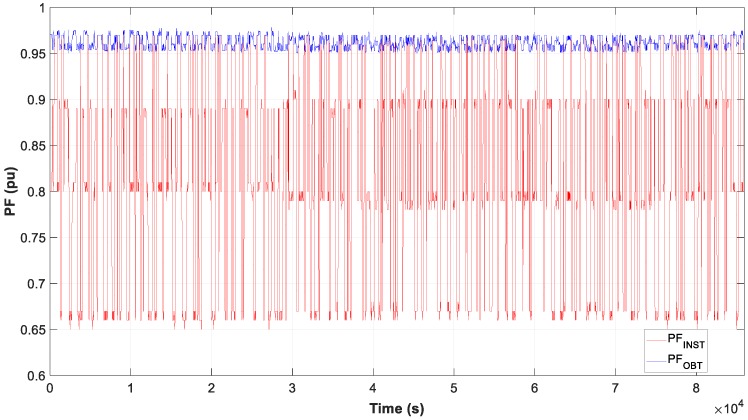
Comparison PF_INST_ vs PF_OBT_ of daily.

**Figure 22:**
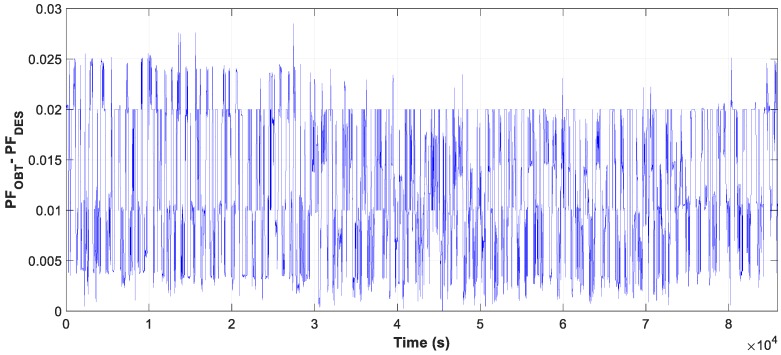
Difference between PF_OBT_ and PF_DES_ of daily.

The result of the measure data is reflected in the three graphs, Figure 20, Figure 21 and Figure 22, the evolution of the obtained vs optimum capacity, PF_INST_ and PF_OBT_, and the difference between PF_OBT_ and PF_DES,_ applying the TLBO algorithm every second.

Figure 21 shows a PF_INST_ greater than 0.95 in some time intervals, with an optimal capacity less than zero and the obtained capacity equal zero, as shown in Figure 20.

The difference between PF_OBT_ and PF_DES_, for induction motors, is less than 0.03. The results obtained with the TLBO algorithm are applicable to these engines.

### 4.4. Cloud Data Logger

The previous tests reflect different time intervals, but in order to obtain the complete monitored of the industry, it is necessary to data stored during the whole period of operation of the equipment.

As an example, this section presents the evolution of the operation of the 1.5 kW motor during one week.

The data stores every second, which coincides with the time of completion of the measures. It is possible to modify the measurement interval depending on the monitoring needs. Also, data is available to download and visualize in the desired time interval.

**Figure 23:**
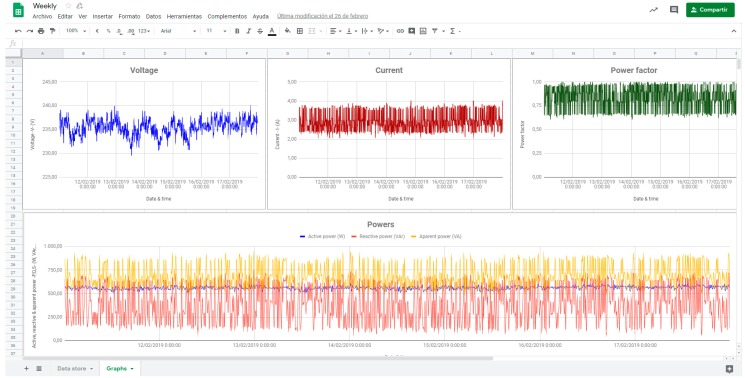
Graphs sheet.

Figure 23 show the graphs in real time depending on the data uploaded during a week in GS.

### 4.5. App in Real Time

Finally, it has been tested the operation making use of the apps developed for Android systems. The application developed makes it possible to monitor the installation via Wi-Fi or 4G connection

The app shows the last 160 values measured by compensation power factor, therefore, taking into account that the measurements are made every second, the last 160 measurements made that will correspond to the last 160 s of operation. In addition to the graphs, the spreadsheet shows the measured instant data

**Figure 24:**
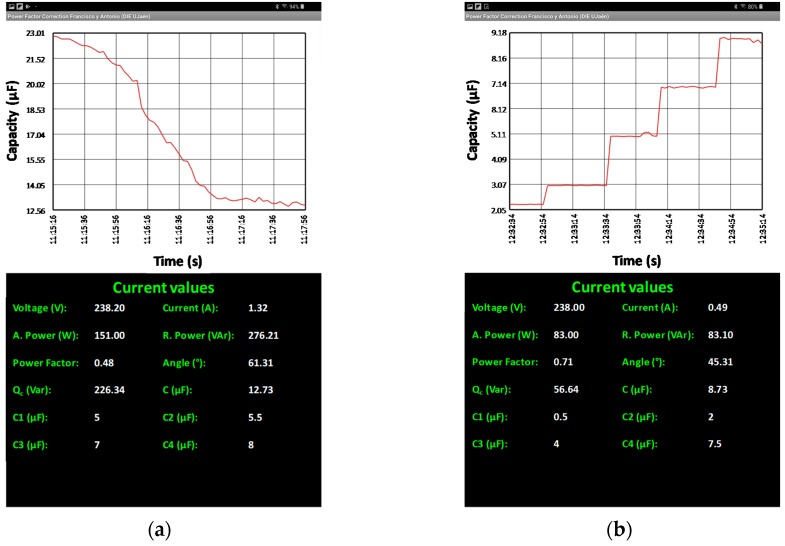
Permanent regime: (**a**) Lamps metal halide; (**b**) Resistance and Inductance Parallel.

The test was done on a Smartphone and Tablet. Following are screenshots of each of the capacity needed and calculated to different loads, shown in Figure 24.

## 5. Conclusions

This project performs the design and implements the compensation power factor using TLBO, determines the best solution by CBO algorithm, and monitors the system in real-time using CDL. Results obtained with the TLBO algorithm, allow us to reduce the processing time of the measured data to achieve the best solution, that determines the switching of capacitors in order to compensate demand of excessive reactive power, thus bringing the power factor near to desired level by means of the CBO algorithm, the values of the optimal capacitors to obtain and install. The data stored in the cloud enables the analysis and improvement of the operation of the installation.

The model of PFCMS shown in this paper allows significant economic savings, both in equipment cost and wiring. That allows visualizing the electrical variables without needing to be present in the place where the measurements are being made.

## Figures and Tables

**Table 1:** Arduino D1R1 characteristics [34].

Parameter	Value
Microcontroller	ESP-8266EX
Operating voltage	3.3 V
Input voltage	9–24 V
Digital I/O pins	11 (provide a PWM output, except D0)
Analog input pins	1
Flash memory	4 MB
Clock speed	80/160 MHz

**Table 2:** Peacefair PZEM characteristics [28].

Parameter	Value
Voltage	80–260 V
Current	0–100 A
Active power	0–22 kW
Energy	0–9999 kWh
Measured signal	Continuous wave
Communication port	TTL port

**Table 3:** Machine characteristics.

Parameter	Squirrel Cage (3 Phase)	Squirrel Cage (3 Phase)	Squirrel Cage (1 Phase)
Rated voltage (Delta-Wye)	230/400 V	230/400 V	230 V
Rate current (Delta-Wye)	5.7/3.3 A	1/0.6 A	2.9 A
Power	1.5 kW	0.37 kW	0.37 kW
Power factor	0.76	0.83	0.96
Speed	1435 rpm	2800 rpm	2870 rpm
Frequency	50 Hz	50 Hz	50 Hz

**Table 4:** Lamps characteristics.

Parameter	Sodium Vapour	Metal Halide
Rated voltage	230 V	230 V
Rate current	1.8 A	1.15 A
Power	150 W	125 W
Frequency	50 Hz	50 Hz

**Table 5:** Resistor and Inductances characteristic.

Position	Value (Ω)	Maximum Power Per Phase (VAr)	Value (H)	Maximum Power Per Phase (W)
1	1050	34	4.46	46
2	750	48	3.19	65
3	435	83	1.84	110
4	300	121	1.27	160
5	213	171	0.9	230
6	150	242	0.64	330

**Table 6:** Comparison of optimization results of capacity for CB.

Type Load	Capacitor Values (μF)	Step	Max. Possibilities	Optimal Solutions	Best Solution C_1_-C_2_-C_3_-C_4_ (μF)
Motor One phase	0.5 … 5	0.5	10,000	9931	0.5-0.5-1-2.5
Motor Three phase Delta	0.5 … 5	0.5	10,000	5770	0.5-0.5-0.5-0.5
Motor Three phase Wye	0.5 … 5	0.5	10,000	4230	0.5-0.5-0.5-0.5
Lamp Metal Halide	0.5 … 10	0.5	160,000	1147	5-5.5-7-8
Lamp Vapour Sodium	0.5 … 10	0.5	160,000	673	5-5-7-8
Lighting Parallel	0.5 … 10	0.5	160,000	4269	4-10-9.5-8.5
Load Resistance/Inductance Series	0.5 … 10	0.5	160,000	3540	0.5-1.5-2.5-2
Load Resistance/Inductance Parallel	0.5 … 10	0.5	160,000	9073	0.5-2-4-7.5
Daily	0.5 … 10	0.5	160,000	23,247	1.5-10-9.5-6.5

**Table 7:** Statistical results of objective function obtained by different loads con TLBO.

Parameter	Mean	Standard Deviation	PF_OBT_ − PF_DES_	
			Max.	Min.
Motor One phase	0.00422	0.00479	0.0174	9.3590 × 10^5^
Motor Three phase Delta	0.00784	0.00852	0.0332	7.7156 × 10^5^
Motor Three phase Wye	0.01669	0.01378	0.0483	7.73490 × 10^5^
Metal Halide	0.01840	0.01058	0.0458	2.90000 × 10^5^
Vapour Sodium	0.01775	0.01107	0.0469	1.2795 × 10^5^
Lighting Parallel	0.00974	0.01172	0.0399	1.0196 × 10^4^
Load Resistance/Inductance Series	0.01313	0.01158	0.0487	6.2545 × 10^7^
Load Resistance/Inductance Parallel	0.01491	0.01625	0.050	9.6650 × 10^5^
Daily	0.01828	0.00686	0.0285	1.3973 × 10^5^

## Abbreviations

The following abbreviations use in this manuscript:

AD1R1	Arduino D1R1
CB	Capacitor Bank
CBO	Capacitor Bank Optimization
CDL	Cloud Data Logger
DL	Data Logger
GS	Google Sheets
IM	Induction Motor
MITAI	MIT App Inventor 2
P	Active Power
PF	Power Factor
PF_DES_	Power Factor Desired
PF_INST_	Power Factor Installation
PF_OBT_	Power Factor Obtained
PFC	Power Factor Correction
PFCMS	Power Factor Compensation and Monitoring System
PLC	Programmable Logic Controller
pop	Population
PZEM	PZEM-004t
Q	Reactive Power
TLBO	Teaching Learning Based optimization
SVC	Static Var Compensator
